# An Improved UU-MESFET with High Power Added Efficiency

**DOI:** 10.3390/mi9110573

**Published:** 2018-11-05

**Authors:** Hujun Jia, Mei Hu, Shunwei Zhu

**Affiliations:** Key Laboratory of the Ministry of Education for Wide Band-Gap Semiconductor Materials and Devices, School of Microelectronics, Xidian University, Xi’an 710071, China; humei@stu.xidian.edu.cn (M.H.); swzhu@stu.xidian.edu.cn (S.Z.)

**Keywords:** 4H-SiC, MESFET, ultrahigh upper gate height, power added efficiency

## Abstract

An improved ultrahigh upper gate 4H-SiC metal semiconductor field effect transistor (IUU-MESFET) is proposed in this paper. The structure is obtained by modifying the ultrahigh upper gate height *h* of the ultrahigh upper gate 4H-SiC metal semiconductor field effect transistor (UU-MESFET) structure, and the *h* is 0.1 μm and 0.2 μm for the IUU-MESFET and UU-MESFET, respectively. Compared with the UU-MESFET, the IUU-MESFET structure has a greater threshold voltage and trans-conductance, and smaller breakdown voltage and saturation drain current, and when the ultrahigh upper gate height *h* is 0.1 μm, the relationship between these parameters is balanced, so as to solve the contradictory relationship that these parameters cannot be improved simultaneously. Therefore, the power added efficiency (PAE) of the IUU-MESFET structure is increased from 60.16% to 70.99% compared with the UU-MESFET, and advanced by 18%.

## 1. Introduction

As a representative of the third generation semiconductor power radio frequency (RF) device, 4H-SiC metal semiconductor field effect transistors (MESFETs) have excellent DC and RF characteristics, such as a high output power density, large saturation current, high breakdown voltage, and large trans-conductance [1,2,3,4,5,6]. Therefore, 4H-SiC MESFETs have great potential in the fields of radars, electronic countermeasures, and other electronic systems. In recent years, many scholars have devoted themselves to studying the direct-current (DC) and RF characteristics of 4H-SiC MESFETs to meet the requirements of the development of electronic science and technology for 4H-SiC MESFETs. However, in response to the national “Energy Conservation and Emission Reduction, the Green Development” call [7,8], to improve the power added efficiency (PAE) of 4H-SiC MESFETs will become a new trend of research and development.

In this paper, an improved ultrahigh upper gate 4H-SiC metal semiconductor field effect transistor (IUU-MESFET) structure with high PAE is proposed based on an ultrahigh upper gate 4H-SiC metal semiconductor field effect transistor (UU-MESFET) [9]. The proposed IUU-MESFET structure achieves high PAE by modifying the ultrahigh upper gate height *h* of the UU-MESFET. This is because increasing the ultrahigh upper gate height *h* can reduce the area of the depletion layer under the ultrahigh upper gate, which not only increases the saturated drain current, but also restrains the expansion of the depletion layer to source/drain sides and reduces the gate-source capacitance. Meanwhile, the enhancement of the ultrahigh upper gate height *h* alleviates the edge effect of the electric field, thereby improving the breakdown voltage. In sum, the ultrahigh upper gate *h* affects the DC and RF characteristics of the device, affecting the PAE of the structure. The IUU-MESFET structure has a larger threshold voltage and trans-conductance, and smaller breakdown voltage and saturated drain current, compared with the UU-MESFET. Additionally, a small threshold voltage absolute value indicates that the IUU-MESFET device is more easily depleted from the steering inversion; a high trans-conductance illustrates that the decrease of the distance between the ultrahigh upper gate and the bottom of the channel makes the gate voltage more capable of controlling the drain current in the channel. In general, the IUU-MESFET balances the size relationship between the structure parameters, so that the structure has high PAE and better DC and RF characteristics. 

## 2. Device Structure 

**Figure 1:**
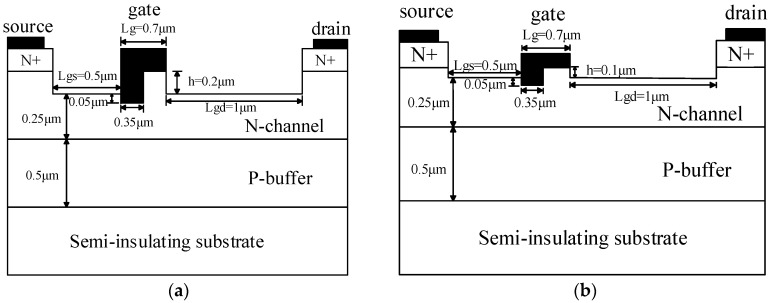
(**a**) Structural cross sections of ultrahigh upper gate 4H-SiC metal semiconductor field effect transistor (UU-MESFET); (**b**) Structural cross sections of improved ultrahigh upper gate 4H-SiC metal semiconductor field effect transistor (IUU-MESFET).

The schematic cross sections of the UU-MESFET and IUU-MESFET structures are shown in Figure 1a,b, respectively. From the bottom to the top of the two structures are, in order, a semi-insulating substrate, a p type buffer with a doping concentration of 1.4 × 10^15^ cm^−3^ and a thickness of 0.5 μm, an n type channel with a doping concentration of 3 × 10^17^ cm^−3^ and a thickness of 0.25 μm, and two highly doped n type cap layers with a doping concentration of 1 × 10^20^ cm^−3^ and a thickness of 0.2 μm. The same dimensions are as follows: gate-source spacing *L_gs_* = 0.5 μm, gate length *L_g_* = 0.7 μm, gate-drain spacing *L_gd_* = 1 μm, the low gate length is 0.35 μm, and the channel is etched 0.05 μm to form the low gate. However, the obvious difference between the UU-MESFET and IUU-MESFET is the ultrahigh upper gate height *h*. The *h* of the two structures is 0.2 μm and 0.1 μm, respectively.

The DC and RF characteristics of the two structures are simulated by the two-dimensional simulation software integrated systems engineering technology computer aided design (ISE-TCAD) based on three basic equations of semiconductors (Poisson equation, electron and hole continuity equation, and electron and hole drift and diffusion equation). In the process of advanced design system (ADS) simulation [10], the eesof scalable nonliear GaAsFet model (EE_FET3) is used because the model satisfies the characteristics of 4H-SiC MESFETs. The modified EE_FET3 model is put into the “Load Pull-PAE, Output Power Contours” of ADS for simulation. Additionally, the working conditions are set as follows: *V_gs_* is 3.2 V, *V_ds_* is 28 V, RF_Req is 850 MHz, Pavs_dBm is 24 dBm, and characteristic impedance *Z*_0_ is 50 Ω. The influence of parameters on PAE is obtained by changing the structural parameters of the device and maintaining the working conditions.

## 3. Results and Discussion

### 3.1. The Influence of Structural Parameters on Power Added Efficiency (PAE)

**Figure 2:**
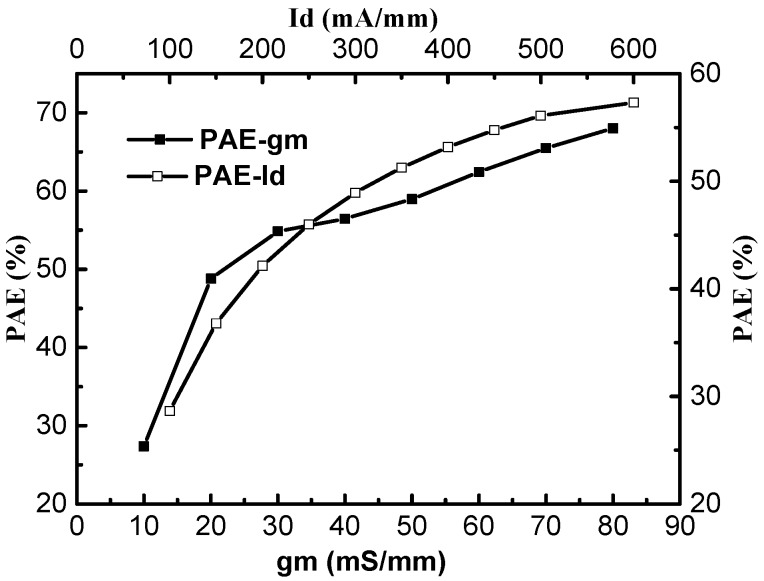
The effect of structural parameters g_m_ and I_d_ on power added efficiency (PAE).

**Figure 3:**
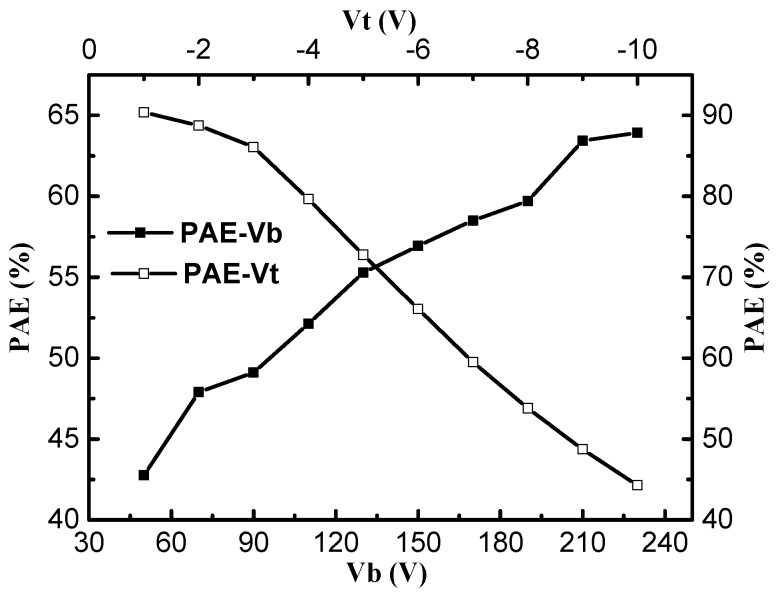
The effect of structural parameters *V_b_* and *V_t_* on PAE.

Figure 2 and Figure 3 show the changes in PAE with the trans-conductance (*g_m_*), the saturation drain current (*I_d_*), the breakdown voltage (*V_b_*), and the threshold voltage (*V_t_*) for the UU-MESFET structure. It is found that improving the trans-conductance, the saturation drain current, the breakdown voltage, and the forward conduction threshold voltage is beneficial to increasing the PAE of the UU-MESFET structure. By comparing the influence degree of structural parameters on PAE, it can be seen that the threshold voltage has the greatest impact on PAE, followed by trans-conductance and the breakdown voltage, and the saturation drain current is the least affected. However, there is a contradiction between the structure parameters of the IUU-MESFET device, and the structure parameters cannot be added at the same time, so a suitable ultrahigh upper gate height *h* is needed to balance these parameters so as to obtain a higher PAE. 

PAE represents the power amplification capability of the device [10]. Its mathematical expression is shown as (1). Furthermore, the maximum output power density for a Class A amplifier is given by expression (2).

(1) $$\;\mathrm{PAE}=\frac{P_{out}-P_{in}}{P_{dc}}\;$$

(2) $$\;P_{max}=\frac{I_{d}\left(V_{b}-V_{knee}\right)}{8}\;$$

where *P_out_* is the output power of the device, *P_in_* is the input power, *P_dc_* is the DC dissipative power, and *V_knee_* is the knee voltage. The combination of expressions (1) and (2) can prove that increasing the saturation drain current and the breakdown voltage of the device can enhance the PAE. However, the mechanism of increasing PAE by raising the forward conduction threshold voltage and trans-conductance remains to be explored. 

### 3.2. Optimization and Analysis of the Device Structure

**Figure 4:**
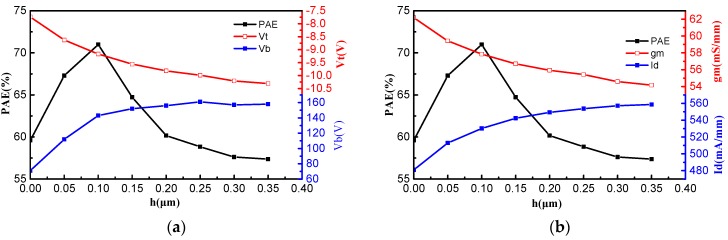
(**a**) The effect of the ultrahigh upper gate height *h* on PAE, $V_{t}$ and $V_{b}$; (**b**) The effect of the ultrahigh upper gate height *h* on PAE, $V_{t}$ and $V_{b}$.

Figure 4a,b show the optimization results obtained by changing the ultrahigh upper gate height *h*. It can be seen that the device has the highest PAE when the ultrahigh upper gate height *h* is 0.1 μm, and with the increase of the ultrahigh upper gate height *h*, the saturation drain current and breakdown voltage first increase, and finally tend to saturate, whereas the trans-conductance and threshold voltage decrease by degrees. Therefore, it is impossible to improve PAE by increasing the breakdown voltage, threshold voltage, saturation drain current, and trans-conductance at the same time.

In order to solve the problem that the structural parameters cannot be increased simultaneously, it is necessary to find a suitable ultrahigh upper gate height *h* to balance the relationship between these structure parameters, so as to obtain a larger PAE. As can be seen from Figure 4, the suitable ultrahigh upper gate height *h* is 0.1 μm. When the ultrahigh upper gate height *h* is 0.1 μm, the device structure has a larger threshold voltage and trans-conductance, and smaller breakdown voltage and saturation drain current, compared with the device structure with an ultrahigh upper gate height of 0.2 μm. Hence, in order to improve the PAE of the UU-MESFET, we must balance the relationship between structural parameters while pursuing the increase of breakdown voltage, threshold voltage, saturation drain current, and trans-conductance.

### 3.3. Discussion of the Device Structure

It can be seen from Figure 4 that the optimal structure known as the IUU-MESFET device is obtained when the ultrahigh upper gate height *h* = 0.1 μm. Table 1 shows the simulation results by ISE TCAD and ADS for the UU-MESFET and IUU-MESFET. According to the Table 1, the PAE values of the two structures are 60.16% and 70.99%, respectively. It can be calculated that the IUU-MESFET structure has an approximately 18% larger PAE value than that of the UU-MESFET structure. Therefore, the IUU-MESFET structure obtains a significant improvement in PAE. 

It can also be found from Table 1 that the UU-MESFET structure has a larger saturation drain current and breakdown voltage, and the IUU-MESFET structure has a greater threshold voltage and trans-conductance. Therefore, from the DC and RF characteristics of the MESFETs, the UU-MESFET structure is a good choice. However, in terms of efficiency, the UU-MESFET structure is the best. This shows that PAE is not always the best when the MESFETs exhibit a good performance.

## 4. Conclusions

In this paper, how to improve the PAE of the UU-MESFET structure has been studied, and the IUU-MESFET structure with high PAE is obtained when the ultrahigh upper gate height *h* is 0.1 μm. The simulation results indicate that improving the threshold voltage, trans-conductance, breakdown voltage, and saturation drain current can increase the PAE of the UU-MESFET. It is found that enhancing the ultrahigh upper gate height *h* can increase the breakdown voltage and saturation drain current, and reduce the trans-conductance and threshold voltage. When *h* is 0.1 μm, the IUU-MESFET structure has a high PAE value and better DC and RF characteristics. The simulation results of ISE TCAD and ADS show that in order to improve the PAE of the UU-MESFET device, the breakdown voltage, the threshold voltage, the trans-conductance, and the saturation drain current should be increased, and the size relationship between them should be balanced. This may serve as a general design direction for improving the PAE of 4H-SiC MESFETs.

## Figures and Tables

**Table 1:** Comparison of structural parameters for the two structures.

Parameter	UU-MESFET	IUU-MESFET
*V_t_* (V)	−9.82	−9.17
*g_m_* (mS/mm)	55.93	57.84
*V_b_* (V)	156	143
*I_d_* (mA/mm)	549.28	530.20
PAE (%)	60.16	70.99
